# Molecular chess? Hallmarks of anti-cancer drug resistance

**DOI:** 10.1186/s12885-016-2999-1

**Published:** 2017-01-05

**Authors:** Ian A. Cree, Peter Charlton

**Affiliations:** 1 Department of Pathology, University Hospitals Coventry and Warwickshire, Coventry, CV2 2DX UK; 2 Faculty of Health and Life Sciences, Coventry University, Priory Street, Coventry, CV1 5FB UK; 3 Imperial Innovations, 52 Princes Gate, Exhibition Road, London, SW7 2PG UK

**Keywords:** Cancer, Chemotherapy, Resistance, Tyrosine kinase inhibitor, Apoptosis, Proliferation, DNA damage, Detoxification, Microenvironment, Heterogeneity

## Abstract

**Background:**

The development of resistance is a problem shared by both classical chemotherapy and targeted therapy. Patients may respond well at first, but relapse is inevitable for many cancer patients, despite many improvements in drugs and their use over the last 40 years.

**Review:**

Resistance to anti-cancer drugs can be acquired by several mechanisms within neoplastic cells, defined as (1) alteration of drug targets, (2) expression of drug pumps, (3) expression of detoxification mechanisms, (4) reduced susceptibility to apoptosis, (5) increased ability to repair DNA damage, and (6) altered proliferation. It is clear, however, that changes in stroma and tumour microenvironment, and local immunity can also contribute to the development of resistance. Cancer cells can and do use several of these mechanisms at one time, and there is considerable heterogeneity between tumours, necessitating an individualised approach to cancer treatment. As tumours are heterogeneous, positive selection of a drug-resistant population could help drive resistance, although acquired resistance cannot simply be viewed as overgrowth of a resistant cancer cell population. The development of such resistance mechanisms can be predicted from pre-existing genomic and proteomic profiles, and there are increasingly sophisticated methods to measure and then tackle these mechanisms in patients.

**Conclusion:**

The oncologist is now required to be at least one step ahead of the cancer, a process that can be likened to ‘molecular chess’. Thus, as well as an increasing role for predictive biomarkers to clinically stratify patients, it is becoming clear that personalised strategies are required to obtain best results.

## Background

Resistance often follows initial responses to chemotherapy. This phenomenon was first noted for alkylating agents in the 1940s [1–3], and although combinations of chemotherapeutic agents led to improved survival [4–6], resistance has remained a problem for classical chemotherapy and newer targeted agents [7]. Cell culture methods allowed study of the phenomenon in vitro, and cell lines have been widely used to explore the mechanisms involved [7, 8]. Classical multidrug resistance (MDR) was recognised early in the development of chemotherapy and *MDR1* (*ABCB1*, PgP) was identified in 1986 [9], followed by other drug efflux pumps [10].

**Fig. 1:**
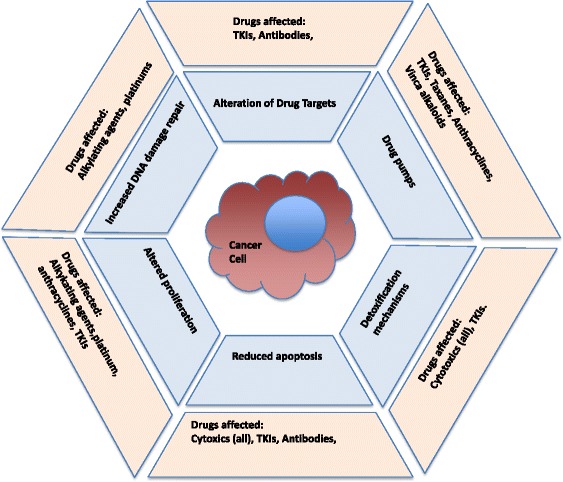
We recognise six hallmarks of anti-cancer drug resistance. Cancer cells may alter drug targets by mutation or reduced expression; upregulate the expression of drug pumps; increase the activity of expression of drug detoxification mechanisms; reduce their susceptibility to apoptosis; alter their level of proliferation; and increase their ability to repair DNA damage. All of these may be employed at once, but there is considerable heterogeneity between tumours, requiring an individualised approach to cancer treatment

The rapidity with which cancer cells can develop resistance to chemotherapy is startling. Using samples from an early neoadjuvant breast cancer trial, we were able to show a considerable difference in chemosensitivity between cancer cells obtained prior to and following four cycles of CMF chemotherapy [11]. The dogma that resistance arose from overgrowth of resistant cell clones due to new mutations was clearly incorrect. We went on to show that tumour-derived cells in primary cell culture down-regulate drug targets and up-regulate resistance mechanisms compared with untreated cells [12]. It is now clear that cancer chemosensitivity is governed by the relative expression of sensitivity and resistance mechanisms, determined by both genetic and environmental factors within tumours [13–15].

Initially many tumours appear to respond to treatment but, as not all the neoplastic cells are killed, this residual population enables regrowth of tumours that no longer respond to a wide variety of drugs [11]. This is cannot be explained by just one mechanism: extreme drug resistance is far more likely to be derived from both gene regulation and mutation. Thus, although in some cases acquired drug resistance may appear to be due to specific mutations, in many cases rapid resistance originates from multiple non-mutational, non-genetic mechanisms [12, 14, 15].

As targeted agents such as tyrosine kinase inhibitors (TKIs) came into practice, it was rapidly noted that these too exhibited the development of resistance, but usually a much slower rate [16–18]. In GIST, imatinib resistance was been found to be due to new mutations, and these often arise in one deposit while others continue to respond [19–24]. Similar results are seen for other mutation-targeted agents including epidermal growth factor receptor (EGFR) inhibitors in non-small cell lung cancer (NSCLC) [25–28], BRAF inhibitors in melanoma [29, 30], and HER2 inhibition in breast cancer [31, 32]. A tumour can compensate for EGFR (HER1) blockade through the activation of alternative signaling pathways such as amplification of MET as well as through changes in tumour microenvironment [33]. EMT has also been reported in NSCLC samples from patients who had developed resistance to EGFR inhibition [33], and some patients develop small cell lung cancer, via neuroendocrine differentiation [34].

## Mechanisms of resistance

The principles underlying the development of anti-cancer drugs resistance apply across all the anti-cancer drugs we have studied, though some are more common in different drug-tumour combinations. The mechanisms fall into a number of distinct categories ﻿(﻿Fig. 1), and often occur together, complicating attempts to combat them:

1. Alteration of Drug Targets: While it is common to separate drugs used in chemotherapy from newer agents targeting molecular pathways, it is of course a truism that all drugs have targets. These targets can be altered by cells in a number of ways. Rapid down-regulation of a target gene expression is an obvious ploy, exemplified by the effect of doxorubicin on topoisomerase IIα [12], but more subtle alteration of drug targets by mutation is also common particularly in response to targeted agents such as receptor tyrosine kinase inhibitors [21, 22, 25, 30, 32]. If the target is part of a pathway activated by other molecules, then the cell may activate an alternative molecular mechanism – mutation of EGFR in ALK fusion gene positive lung cancer is a good example [28, 35, 36].
2. Expression of drug efflux pumps: The ATP-binding cassette (ABC) superfamily of proteins includes a number of membrane proteins able to transport a wide diversity of substrates. Besides an ability to transport of toxins out of cells, other substrates include amino acids, peptides, sugars, lipids, steroids, bile salts, nucleotides and endogenous metabolites [10]. These pumps act to protect cells by ejecting a wide variety of toxins. Although in bacteria this toxin might be an antibiotic, in human cancer it is often an anticancer drug. Classical drug resistance is mediated by the *MDR1* (*ABCB1*) gene, which encodes a membrane-based xenobiotic pump molecule, known as phenolic glycoprotein (PgP). This pump is relatively promiscuous and ejects drugs from the cell at a rate that may exceed their entry, rendering the cell resistant. One of the more important molecules of the blood-brain barrier, it has been much studied. This in turn led to the discovery of numerous other pumps, and the human genome contains 49 ABC transporter molecules [10], many of which can pump drugs. Besides MDR1 the best known are multidrug resistance related protein (*MRP1, ABCC1*) and breast cancer related protein (*BCRP, ABCG2*). Pharmaceutical chemists now design drugs with this mind, so that pump mechanisms are less problematic than they were, though even some TKIs, including gefitinib and erlotinib [37, 38], are pumped. Metabolite and nucleotide pumps have also been found to be of importance, and genes such as hENT1 have been reported to be important mediators of chemosensitivity in gene expression studies [13–15]. Rapid up-regulation of drugs pumps can occur in cancer cells and lead to resistance [12].
3. Expression of detoxification mechanisms: Drug metabolism occurs at the host level, where it underlies the pharmacokinetics of many drugs, and within cancer cells themselves, where there may be considerable heterogeneity. Molecules such as gluthathione S-transferase (GSTπ) are well known to be up-regulated in some cancers and a potential cause of resistance [12, 39]. It is possible that conjugation and excretion of drugs at the luminal surface of some well-differentiated adenocarcinomas may explain the relationship between differentiation and drug sensitivity to some drugs, but this remains uncertain [40–42]. Altered local drug metabolism and detoxification are key resistance mechanisms across many cancers. As an example, these processes have been investigated in the plasma cell cancer, Multiple Myeloma (MM), where a majority of patients repeatedly relapse and finally succumb to the disease [43]. The expression of 350 genes encoding for uptake carriers, xenobiotic receptors, phase I and II drug metabolising enzymes and efflux transporters was assessed in MM cells of newly-diagnosed patients. There was a global downregulation of genes encoding for xenobiotic receptors and downstream detoxification genes in patients with an unfavourable outcome. However, there was a higher expression of genes encoding for the aryl hydrocarbon receptor nuclear translocator and Nrf2 pathways as well as the ABC transporters in these patients [43].
4. Reduced susceptibility to apoptosis and cell death: Apoptosis was recognised as a unique form of cell death by Currie and others in the 1970s [44, 45]. It attracted the attention from pathologists, but it was not until experiments by Gerard Evan et al. [46–48] that it became clear that avoidance of apoptosis underpinned the development of cancer and was an important resistance mechanism for cancer cells to both chemotherapy [8, 47, 48] and agents targeting signaling pathways [49–51]. Other forms of cell death may also be triggered by anti-cancer drugs, including necrosis, necroptosis, and autophagy [52]. In all cases, the key feature in resistance seems to be survival signalling which prevents cell death. Not all forms of cell death are the same, and the level of damage required to achieve cell death is variable. This particularly true of autophagy, which can either promote chemosensitisation or chemoresistance [53]. In some cases, its inhibition can chemosensitise tumours [54]. Necroptosis is a caspase-independent form of cell death induced by receptor-interacting protein kinases (RIP1 and RIP3) or mixed lineage kinase domain-like protein (MLKL). Its importance in cancer treatment is controversial, but its induction may circumvent anti-apoptotic mechanisms [55].
5. Increased ability to repair DNA damage: As cancers must acquire permanent genomic mutations, cancer can be viewed as a disease of DNA repair as changes in these genes produce the mutator phenotype essential the for the acquisition of further mutations. Once a mutation is acquired cancers often become addicted to a different DNA repair pathway. A good example of this is exemplified by BRCA1/2. As BRACA1/2 are key components of a DNA double strand repair pathway these cancers become dependent on another DNA repair component, PARP1, for replication fork progression [56, 57]. Inhibition of PARP1 in these cancer cells is catastrophic and results in their death. This is the concept of synthetic lethality [58] and has been proposed as a potential Achilles heel in a cancer cell’s defense. Although this concept may enable the clinician to increase the therapeutic index between cancer and normal cells, it is expected that these approaches will also have the potential to develop resistance. DNA damage is recognised by cells, and if they cannot repair the damage, this leads to apoptosis [12, 59]. If apoptotic potential is reduced, then cells can survive considerable DNA damage, but an alternative is to up-regulate DNA repair [59]. Many cells of course do both.
6. Altered proliferation: The normal response to DNA damage that cannot be repaired is apoptosis, but as Gerard Evan showed in diploid fibroblasts, the threshold for death is much higher in cells that are not growing [46]. Transient reduction in growth is mediated in part by P53 [60]. Levels of P53 rise and at first simply reduce cell cycle, only tipping over to stimulate apoptosis at a certain threshold [60].

## Other factors

In addition to these key mechanisms there are several other factors external to the neoplastic cell that can contribute to resistance. These include the influence of the tumour stroma and microenvironment [61, 62], the role of tumour-initiating cells as part of intra-tumoural heterogeneity, autophagy [63] and intra-tumoural heterogeneity [64].

Although much of the field of cancer drug resistance has been focused on the tumour cell and its ability to develop resistance, the ‘host’ can also play a major role in promoting resistance to therapy. Chemotherapy, targeted therapies and radiotherapy all lead to the recruitment of different host cells and factors to the tumour microenvironment. This host response can then contribute to resistance by inducing various cellular and molecular pathways that support the tumour, for example by driving tumour cell proliferation (Altered Proliferation) or survival (Reduced susceptibility to apoptosis). As the tumour microenvironment is heterogeneous it can help support a variety of resistance mechanisms. For example various cellular pathways are affected by tumour hypoxia, and thus tissue hypoxia can contribute to development of resistance [61].

Tumour-initiating cells may also play a key role in tumour recurrence as these cancer stem cells (CSCs) are able to utilise a number of mechanisms to evade chemotherapy (e.g. expression of ABC transporters, enhanced expression of aldehyde dehydrogenase, expression of pro-survival proteins altered DNA damage response and altered signaling pathways) [65, 66]. As a result of their ability to better tolerate drug exposure, CSCs are often refractory to drug treatment. For example, integrin αvβ3 expression in multiple types of solid tumour stem cells controls a pro-survival pathway involving activation of KRAS, which may contribute to TKI drug resistance [67].

Autophagy is a complex issue as induction of autophagy can have both a pro-death and a pro-survival role [63, 68]. Thus autophagy can influence both the anticancer efficacy of drugs as well as contribute to drug resistance. In its role as a cellular housekeeper autophagy removes damaged organelles and by recycling macromolecules can protect against cancer formation. However, in established tumours this ‘protective’ role can switch to a pro-survival function as, when cancer cells are stressed, autophagy can enable the tumour to respond to its environmental conditions through reducing growth and by increasing the catabolic turnover of unnecessary proteins and organelles. Under these conditions inhibition of autophagy may actually help overcome resistance to chemotherapy [68].

Tumours show considerable heterogeneity in the extent to which they use these mechanisms [12, 15]: and it is likely that there is also considerable intra-tumoural heterogeneity, given the presence of multiple clones within tumours [64].

## Use of multiple mechanisms

Cancers can employ several resistance mechanisms, either sequentially or concurrently to evade drug treatment. Four examples are described to illustrate this point, including classical chemotherapy and targeted agents:

1. Topoisomerase II inhibitors remain a mainstay of both haematological and solid tumour therapy, but their clinical efficacy is often limited by resistance. Many mechanisms may contribute to this resistance including reduced drug accumulation and/or increased efflux, site specific mutations affecting drug-induced topo II mediated DNA damage, post-translational modifications resulting in altered DNA damage and downstream cytotoxic responses [12, 15].
2. Anti-HER2 antibodies such as Herceptin develop acquired resistance through a variety of mechanisms, including activation of tyrosine kinase in CSCs, upregulation of HER3, activating mutations in the p110a subunit of PIKK (PIK3CA), enhanced HER-ligand autocrine signaling and alterations in apoptotic pathways [69]. HER3 has now also been proposed as potentially driving survival of HER2+ cells once they have developed resistance to HER2 inhibitors such as lapatinib and trastuzumab [69].
3. Bortezomib was the first proteasome inhibitor to enter practice. Again a wide range of mechanisms have been reported in acquired resistance to this drug, which has an important role in the treatment of several haematological cancers. These include mutations in proteasome subunits, unfolded protein response, XBP1 and MARCKS proteins, aggresomes, the role of constitutive and immunoproteasomes, alterations in pro-survival signalling pathways, changes in bone marrow microenvironment and autophagy as well as other multidrug resistance mechanisms [70, 71].
4. Antibody-drug conjugates can also be limited by acquired resistance [72]. As with small molecules, this resistance is multifactorial and can include altered interaction with the target, altered apoptosis pathways and altered survival pathways. In addition, the payload for any antibody drug conjugate approach is likely to be sensitive to the same range of resistance mechanisms described for small molecule drugs.

It is clear that understanding these mechanisms has enabled the field to undertake more rational development of the next generation of drugs to overcome clinical resistance. Beyond chemical modification of a drug, advances in other technologies are also looking promising. For example nanoparticle delivery systems to allow better targeting or addressing specific molecular alterations in resistant tumours [73]. It may also be possible to develop multifunctional nanoparticles able to simultaneously target multiple resistance mechanisms.

## Strategies to overcome resistance

Understanding of resistance mechanisms has now advanced to the point where experimental approaches can now start to predict clinical drug resistance. In vitro these approaches include target-based mutagenesis, use of isogenic tumour cell lines, both gain and loss of function resistance screens, and in depth analysis (cellular, genomic and molecular) of drug-resistant tumours. Further information will be gained from both genetically-engineered mouse models, patient-derived xenografts, and *ex vivo* primary cell culture models. A number of strategies have been used in cancer treatment to overcome the problem of resistance.

1. The development of new synthetic analogues of existing drugs has been the usual response to try to circumvent resistance. It is possibly best exemplified in the vinca alkaloid derived drugs, where greater potency has been achieved by chemical alteration of molecules [74, 75]. In some cases however, this approach has been less than successful, as it tends to increase toxicity.
2. Combinations have been used in oncology since multiple drugs became available. Most combinations have been developed empirically, on the basis that if two drugs are active, then the combination should be more active still. This has been a successful approach, but as the number of possible combinations has risen, the number of expensive clinical trials required to fine-tune such combinations has made this approach less attractive. Cell lines have been used to design combinations, with some success, but the reality is that highly passaged cell lines are poor models of cancer cell behavior [76, 77]. We have previously used primary cell culture to develop new combinations, with considerable success [78].
  It is clearly important to stratify patients based on whether they are likely to respond to a particular therapy or combination. Although cell lines can provide a useful first step they are unable to effectively model the complex tumour–stroma interactions that contribute to the development of drug resistance. It is now suggested that combining therapies that target two or more orthogonal, ‘independent’ pathways, will be preferable to attempting to hit two or more targets on the same pathway. It is hoped that this approach will reduce the tumour’s ability to mount an effective resistance campaign.
3. Sequential strategies have much to recommend them, both to increase efficacy and reduce toxicity. Despite some success, relatively few sequential combinations have entered clinical practice, as until recently the molecular understanding of their efficacy has been lacking [79]. DNA and RNA sequencing technologies are now at a point where they can be used as companion diagnostic technologies, and the effects of sequential drug administration can be predicted [80].
4. Synthetic lethality is used to describe a mechanistic approach to combination and sequence design. Tumour-specific genetic changes can make cancer cells more vulnerable to synthetic-lethality strategies and so enable the clinician to target tumour cells while sparing normal cells. These mutations in cancer genes may be either loss or gain of function and the concept can be extended to contextual synthetic lethality to include defects in metabolic processes and rewiring signaling networks and tumour-associated hypoxia [81]. Nevertheless, even with a new generation of novel targeted cancer therapies based on the concept of synthetic lethality, the potential for secondary acquired resistance remains. Mutation or inactivation of P53 is usually thought to be anti-apoptotic, allowing cells to avoid the induction of apoptosis. However, chemosensitivity experiments in ovarian cancer showed that this was not always the case [82], and subsequent studies have shown that under certain conditions, mutation of P53 can confer susceptibility to apoptosis [60]. It is increasingly clear that such approaches to synthetic lethality are achievable with sufficient knowledge of the molecular makeup of individual cancers [60]. In high grade serous ovarian cancer, characterised by P53 mutation, 20% of patients have BRCA1 and BRCA2 mutations rendering them susceptible to PARP inhibitors, and methylation of the BRCA1 promoter has a similar effect [83]. Drug development and companion diagnostic development strategies need to be aligned, and tested in a variety of pre-clinical settings before use in man.
5. Immunotherapy has long been suggested as a solution to many of the problems of anti-cancer drug resistance. The advent of anti-CTLA antibody treatment with ipilimumab in melanoma [84] showed that the promise was likely to be fulfilled, and the success of anti-PD1 and anti-PDL1 antibodies alone and in combination with anti-CTLA4 antibody treatment is nothing short of a revolution in melanoma and lung cancer, to mention but two target cancers of the many that are likely to benefit from these agents. Understanding of resistance to these agents is at an early stage [85], and the benefits of combination or sequential use of immunotherapeutics with chemotherapy or targeted agents has yet to be established. However, it is already clear that PDL1 expression by the neoplastic cells is useful as a companion diagnostic, despite difficulties of implementation [86]. Neo-antigen load is related to mutational load [87], and cancers with high mutational load seem to respond well to immunotherapy [88, 89]. It is entirely possible that accurate immune profiling of tumours will require multiple methods.

Genetic and epigenetic events, as well as extracellular signals, can activate pathways that enable cancer cells to become chemoresistant to therapeutic agents. This situation has encouraged a more systematic approach to identifying those signaling pathways that might confer resistance to cancer drugs. This approach will not only help stratify patients into groups either more or less likely to respond, but also will help design drug combinations that act simultaneously on multiple cancer cell dependencies and resistant pathways.

## Molecular pathology

Alterations of proteins and nucleic acids can be identified with increasing accuracy, and their concentration measured accurately and precisely using a variety of different methods [90, 91], some of which can be applied to blood samples without ever needing a biopsy of the tumour. Implementation of these methods in pathology departments is proceeding rapidly [90], and the measurements are increasingly used by oncologists to tailor treatment to individual measurements. The challenge to the diagnostic pathologist is to go beyond diagnosis to provide the information needed to treat the patient [92], while the challenge to the oncologist is to understand the information provided and adopt a strategy that gives the patient the longest possible survival with the greatest possible quality of life [93, 94]. The use of specific TKIs is often guided by closely linked companion diagnostics, such as EGFR or KRAS mutation status according to the licensed indication [92]. Late resistance mechanisms often involve further mutations, and these may require more extensive testing. In contrast to companion diagnostics, the term ‘complementary diagnostics’ describes a broader group of diagnostics associated with a class of drugs, unconstrained by drug license and used to guide therapy [95]. As the number of drugs and targets increase, use of complementary diagnostics are likely to be required to optimise therapy, based on methods such as targeted next generation sequencing [80].

## Conclusions

Cancer teams need to learn to play molecular chess – effectively outthinking the cancer’s likely response to any treatment used, and to be ready for it. The tools provided by the pharmaceutical industry to allow this have never been better, and coupled with increasingly sophisticated radiotherapy and surgery, allow many patients to survive for years and even decades with cancers that would have killed them rapidly just 30 years ago. The principles of drug resistance – or perhaps the rules of molecular chess – are increasingly clear and can improve patient care.

## Abbreviations

ABC: ATP-binding cassette
*ABCB1*: ATP binding cassette subfamily B member 1
ALK: Anaplastic lymphoma receptor tyrosine kinase
ATP: Adenosine triphosphate
BCRP: Breast cancer related protein (*ABCG2*)
BRAF: B-Raf proto-oncogene, serine/threonine kinase
BRCA: BRCA, DNA repair associated
CMF: Cyclofosfamide, Methotrexate and 5-Fluorouracil
CSC: Cancer stem cell
CTLA4: Cytotoxic T-lymphocyte associated protein 4
DNA: Dexoxyribose nucleic acid
EGFR: Epidermal Growth Factor Receptor
EMT: Epithelial mesenchymal transition
GIST: Gastrointestinal stromal tumour
GSTπ: Gluthathione S-transferase
HER: Human epidermal growth factor receptor
KRAS: Kirsten rat sarcoma viral oncogene homolog
MARCKS: Myristoylated alanine rich protein kinase C substrate
MDR: Multidrug resistance (MDR)
MET: MET proto-oncogene, receptor tyrosine kinase
MLKL: Mixed lineage kinase domain-like protein
MM: Multiple Myeloma
MRP1: multidrug resistance related protein (*ABCC1*)
Nrf2: Nf-E2 related factor 2
NSCLC: Non-Small Cell Lung Cancer
P53: Tumor protein p53
PARP1: Poly(ADP-ribose) polymerase 1
PD1: Programmed cell death protein 1
PDL1: Programmed death ligand 1 (B7-H1, CD274)
PgP: p-glycoprotein
PIK3CA: p110a subunit of PIKK
RIP1: Receptor-interacting protein kinase 1
RIP3: Receptor-interacting protein kinase 3
RNA: Ribonucleic acid
TKI: Tyrosine kinase inhibitor
XBP1: X-box binding protein 1
